# Stereotactic Body Radiosurgery for Spinal Metastatic Disease: An Evidence-Based Review

**DOI:** 10.1155/2011/979214

**Published:** 2011-07-10

**Authors:** William A. Hall, Liza J. Stapleford, Costas G. Hadjipanayis, Walter J. Curran, Ian Crocker, Hui-Kuo G. Shu

**Affiliations:** ^1^Department of Radiation Oncology, Winship Cancer Institute, Emory University, 1365 Clifton Road NE, Suite CT-104, Atlanta, GA 30322, USA; ^2^Department of Neurosurgery, Winship Cancer Institute, Emory University, Atlanta, GA 30322, USA

## Abstract

Spinal metastasis is a problem that afflicts many cancer patients. Traditionally, conventional fractionated radiation therapy and/or surgery have been the most common approaches for managing such patients. Through technical advances in radiotherapy, high dose radiation with extremely steep drop off can now be delivered to a limited target volume along the spine under image-guidance with very high precision. This procedure, known as stereotactic body radiosurgery, provides a technique to rapidly treat selected spinal metastasis patients with single- or limited-fraction treatments that have similar to superior efficacies compared with more established approaches. This review describes current treatment systems in use to deliver stereotactic body radiosurgery as well as results of some of the larger case series from a number of institutions that report outcomes of patients treated for spinal metastatic disease. These series include nearly 1400 patients and report a cumulative local control rate of 90% with myelopathy risk that is significantly less than 1%. Based on this comprehensive review of the literature, we believe that stereotactic body radiosurgery is an established treatment modality for patients with spinal metastatic disease that is both safe and highly effective.

## 1. Introduction

Spine column tumors, both primary and metastatic lesions, are quite often seen in cancer patients. For a variety of tumors, the spine is the most common site of metastatic disease. It is estimated that 20,000–25,000 patients per year in the US develop spinal cord or root compression as a manifestation of their metastatic disease [1, 2]. Further estimates conclude that 5–10% of cancer patients will develop spinal metastasis [3]. In cancer patients with acute onset of back pain or other clinical suspicion for spinal metastatic disease, rates of spinal metastasis exceeding 25% have been reported [3, 4]. Radiotherapy has long been established as an effective treatment modality for spinal tumors [5–8]. With the advancement of image-guided radiation therapy technology, extracranial spinal radiosurgery has emerged as an effective and safe treatment modality for spinal tumors, both primary and metastatic. 

Extracranial radiosurgery, or stereotactic body radiosurgery (SBRS) was developed in the mid 1990s at various institutes around the world. Possibly the earliest experience describing the procedure came from the Karolinska Institute in Sweden [9]. Around the same time, Hamilton et al. published their early experience treating spinal tumors with linear accelerator-based radiosurgery in the setting of failure of other surgical or radiotherapy interventions [10]. Since the early days of SBRS development, the technique has become increasingly important and common in the management of both primary and metastatic spinal tumors. Today, stereotactic radiosurgery has come to be defined as “a distinct discipline that utilizes externally generated ionizing radiation in certain cases to inactivate or eradicate (a) defined target(s) in the head or spine without the need to make an incision. The target is defined by high-resolution stereotactic imaging [11].” The purpose of this review is to summarize the growing body of literature for spine radiosurgery focusing on prospective case series available that have led to the current standards, and will influence future directions in spinal radiosurgery.

## 2. Technical Aspects of Spine Radiosurgery

**Figure 1:**
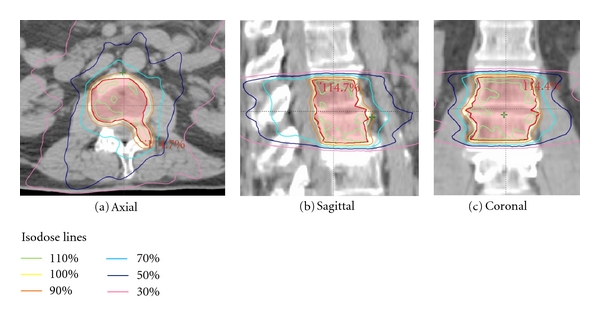
Dose distribution for a patient treated with spinal radiosurgery to L1-L2. The planning target volume (PTV) is outlined in red.

Essential to the delivery of stereotactic radiation to the spine is a very steep dose gradient outside of the target volume [12]. An example of a spine radiosurgery plan for a patient with this type of steep dose fall off and conformal dose distribution is shown in Figure 1. Equally important to the dosimetric considerations is rigid immobilization of the patient. The essential elements required for spinal radiosurgery can be achieved through different commercially available, turn-key or institution-specific, in-house systems. Currently, several available systems, each utilizing slightly different immobilization techniques and methods for accurately delivering focused spinal radiation doses, can be used for this purpose. Some of the more common systems are detailed below. 

The CyberKnife, (Accuray, Inc., Sunnyvale, CA) is a frameless robotic radiosurgical system that is used to deliver extracranial radiosurgery and plays an important role in spinal radiosurgery. The design of the CyberKnife consists of a lightweight linear accelerator (LINAC) mounted on a robotic manipulator that serves to deliver several independently targeted (nonisocentric) and noncoplanar treatment beams. These beams are delivered under continual X-ray image guidance with corresponding shifts in the positioning of the robotic arms to maintain accurate targeting [13]. Early versions of the CyberKnife system required implanted radiopaque markers that were used to accurately localize the spinal target. Recent advancements in the ability of the Cyberknife technology to track the spine (a tracking system called Xsight, Accuray, Inc.) have eliminated the need for implanted fiducials. The treatment positioning for this system compared with use of implanted radioopaque fiducials was found to be 0.61 mm ± 0.27 mm as measured in a a realistic, anthropomorphic head-and-neck phantom and 0.49 mm ± 0.22 mm in 11 patients treated with SBRS [14]. Typical immobilization devices used for the Cyberknife system consist of head thermoplastic masks for cervical spine tumors, and body alpha cradles for thoracic and lumbar tumors. 

A second major commercially available system used to perform both cranial and extracranial radiosurgeries is the Novalis LINAC (BrainLAB, Inc., Munich, Germany). The device consists of a single-energy 6 MV LINAC mounted on a standard gantry with a built-in micromultileaf collimator. As a consequence of being single energy, this treatment unit has a lower mass than typical general purpose LINAC's thus facilitating gantry isocentricity [15]. Similar to the Cyberknife, the Novalis system is equipped with in-room kilovoltage X-ray imaging equipment consisting of two orthogonally mounted 80–100 kiloelectron volt (keV) X-ray tubes with corresponding amorphous silicon digital detectors and a computerized control and image analysis system. The acquired keV images are then fused with the reference images from the CT simulation to ensure accurate patient positioning. The information regarding the location of the isocenter is forwarded to the ExacTrac system, a computerized system that uses two infrared cameras to detect infrared-sensitive markers. This allows the system to automatically compare this marker information with reference information to move the treatment couch to the desired position [16, 17]. The precision for the Novalis system, which was defined on this study as the degree of isocenter variation from CT simulation to portal imaging at the time of treatment, has been measured at 1.36 mm ± 0.11 mm [16]. Both the value measuring variability of the Novalis ExacTrac and the values from the previous paragraph measuring variability of the Cyberknife Xsight show that each system is highly precise. However, their absolute values cannot be directly compared since the gold standard for patient positioning in each study was different (image fusion of digitally reconstructed radiographs (DRRs) from the simulation CT with orthogonal port films for ExacTrac and matching positions of 4 fiducial markers on DRRs with orthogonal X-rays). 

In addition to the above dedicated radiosurgery systems, modern linear accelerators equipped with image-guidance hardware such as the Trilogy system (Varian Medical Systems, Inc., Palo Alto, CA) or the Synergy system (Elekta AB, Stockholm, Sweden) can be used for spinal radiosurgery. Given our experience with the Trilogy, we will discuss that system in greater detail. Like the above systems, the Trilogy utilizes the dosimetric advantage of multiple noncoplanar treatment beams [18]. It is the angular distribution of these beams that enables a conformal dose distribution around a nonspherical target. With this system, the user can select either dynamic mode (also known as sliding window) or segmental mode (also known as step and shoot) to deliver an intensity modulated treatment plan. Each of these treatment modes has their advantages that have been previously described [19–21]. With regards to patient immobilization, any number of solutions can be adapted for use with the Trilogy. For treatment in the mid-lower thoracic and lumber regions, we use BodyFIX (Elekta AB) which consists of a vacuum bead cushion that is set to conform to the patient's treatment position with an overlying plastic wrap that is affixed under vacuum suction over the patient to ensure reproducible setup and reduce potential motion. For treatment in the lower cervical and upper thoracic spine, we use a customized head and shoulder thermoplastic mask with body immobilization. Finally, for treatment in the upper cervical spine, we use a thermoplastic mask that is fitted over the head on an indexed head extender that permits adjustments in all six degrees of freedom (3 translational and 3 rotational). Central to any spinal radiosurgery system is the image guidance system used to confirm patient setup and tumor location with normal anatomical landmarks. The Trilogy features three different imaging/localization systems on the treatment machine, namely, optical, kilovoltage X-rays, and megavoltage X-rays. While each of these image modalities have their advantages and can be used to guide patient positioning either alone or together depending on the situation, we primarily use the kilovoltage on-board imager to obtain both paired orthogonal images and cone-beam CT images for verification of positioning. The kilovoltage imager for Trilogy is mounted on the Trilogy gantry with 2 robotically controlled arms that each operate on three axes of motion which enables optimal positioning for imaging of the target volume [18]. For patient setup, paired orthogonal images are first obtained using this system to guide initial positioning. Next, this same imager is used to obtain a 3D cone-beam CT image set which can subsequently be matched to the simulation CT scan using either automated or manual image registration. A second cone-beam CT scan may then be obtained to confirm final positioning before patient treatment. Beam shaping with the Trilogy is achieved with the 120-leaf Millennium multileaf collimator (MLC). The Millennium MLC has been previously described and its advantages with respect to beam penumbra are established [22, 23]. Finally, the Trilogy features dual beam energies (6 and 18 MV photons) providing greater flexibility in the type of treatment plans that can be used. These features combine to make the Trilogy a versatile machine that is well suited for spinal radiosurgery.

## 3. Dosimetric Considerations in Spinal Radiosurgery

The safety of any course of radiotherapy is dependent on the tolerance of the normal tissues in the vicinity of the tumor that is being treated. Of paramount importance when considering spinal radiosurgery is the dose to the spinal cord. Classically, the tolerance of the spinal cord, according to Emami and colleagues, is expressed in terms of TD 5/5 (tolerant dose of radiation, dose at which the severe complication rate is 5% at 5 years) and is estimated at 50, 50, and 47 Gy for cord lengths of 5, 10, and 20 cm, respectively, for a conventionally fractionated course (1.8–2.0 Gy/fraction) [24]. An important consideration for this report is that its conclusions were based on extrapolation of data going back to the 1940s. In the setting of modern day conformal radiotherapy technologies, many view the stated tolerance of the spinal cord of 45–50 Gy as conservative. More recent data has supported the possibility of a higher spinal cord tolerance [25–29]. In particularly, Kirkpatrick et al. showed that for patients treated with conventional fractionation, the risk of myelopathy is less than 1% at 54 Gy and less than 10% for 61 Gy [29]. 

When discussing spinal radiosurgery, the spinal cord tolerance to hypofractionated RT becomes more important than the spinal cord tolerance to conventionally fractionated radiation (1.8–2.0 Gy). Some information regarding the dose tolerance of the spinal cord to high-dose radiation fractions has emerged. It is well established that common hypofractionation schemes in the dose range of 8 Gy × 1 fraction to 4 Gy × 5 fractions is safe with essentially 0% risk of radiation myelitis [30, 31]. Macbeth and colleagues estimated the risk of radiation myelopathy based on information from three randomized trials of palliative radiotherapy for nonsmall cell lung cancer [28]. According to their review, none of the 114 patients treated with 10 Gy × one fraction developed spinal myelopathy. However, of 524 patients treated with 17 Gy in two fractions, the estimated cumulative risk of myelopathy at 2 years was 2.2%. Additionally, prospective data suggest that the spinal cord can tolerate at least 10 Gy to 10% of this volume when defined as the cord at the level of the radiosurgical target plus 6 mm above or below this region, with acceptable rates of myelitis [32]. 

Important to consider also is the issue of reirradiation of the spinal cord after a fractionated course of RT. While data with respect to cord reirradiation is limited, this question has been examined in a primate model by Ang and colleagues [33]. In their study, a group of 56 rhesus monkeys were initially treated to a dose of 44 Gy in 2.2 Gy fractions to the cervical and upper thoracic spinal cord. Monkeys were then reirradiated using 2.2 Gy fractions to 57.2 Gy after 1 or 2 year intervals or 66 Gy after 2 or 3 year intervals. In this long-term experiment, 45 monkeys completed the required observation period of 2–2.5 year after reirradiation (for a total of 3–5.5 years total followup). Of these monkeys, only 4 developed myeloparesis. The authors concluded that spinal cord tissue likely has a large capacity to recover from prior radiation doses. Some data is also available in regards to reirradiation of the human spinal cord. One clinical series reported on a total of 62 patients reirradiated for an in-field recurrence of spinal cord compression from metastatic disease with 8 Gy × one fraction or 3 Gy × 5 fractions after initially being treated with 8 Gy × one fraction or 4 Gy × 5 fractions. This approach results in a biologically equivalent dose (BED) of 80–100 Gy (by standard linear-quadratic modeling) to the spinal cord and, at a median of 8 months of followup, there were no cases of radiation myelopathy observed [34–36]. Higher incidences of myelopathy have been reported in patients receiving higher BEDs to the cord. In a series of 40 patients reported by Nieder et al., myleopathy was only observed in patients receiving higher than 102 Gy of cumulative BED with no observed cases of myleopathy below that dose [37]. In a recent analysis by Sahgal et al., the dosimetric data in five cases of myelopathy was analyzed per the BED and these were compared to a subset of 19 patients with no radiation myelopathy [38]. The thecal sac was contoured to represent the spinal cord, and doses to a maximum volume of 0.1, 1, 2, and 5 cc were analyzed. Radiation myelopathy was found to occur with a maximum point dose of 14.8, 13.1, and 10.6 Gy in a single fraction, 25.6 Gy in two fractions, and 30.9 Gy in three fractions. The authors concluded from their series that for single fraction SBRT, a maximum point dose of 10 Gy is safe. It should be noted that the data regarding spinal cord tolerance in the setting of reirradiation is still limited and should be clinically applied with caution.

## 4. Selection of Case Series

PubMed, a service of the US National Library of Medicine, was searched for English language publications up through December, 2010 on stereotactic radiosurgery for spinal tumors. Radiosurgery was defined as 5 or fewer fractions of radiation delivered to both primary and metastatic spinal tumors. Treatment in the primary and reirradiation setting were both included in this review. To evaluate only more sizeable experiences, series that had fewer than 20 patients were excluded. A total of fifteen series were identified that met these criteria, and details about these reports are summarized on Table 1.

## 5. Review of the Literature

In a phase II trial from the University of Florida by Amdur and colleagues, 21 patients were treated with a single fraction of 15 Gy with spinal cord dose limited to 12 Gy to no more than 0.1 cc in previously unirradiated patients and 5 Gy to no more than 0.5 cc in previously irradiated patients [39]. A primary objective of this study was to evaluate toxicity. The authors demonstrated that with these dose constraints, patients experienced only minor grade 1-2 acute toxicities consisting primarily of nausea or dysphagia and no late toxicities. Overall, this series demonstrated that, with clearly defined spinal cord dose constraints, spinal radiosurgery given as 15 Gy in a single fraction is very well tolerated. 

Gerszten et al. reported on multiple case series from the University of Pittsburgh about the safety and effectiveness of spinal radiosurgery in patients with different types of metastatic lesions [40–44]. In 77 patients with metastasis to the spine from nonsmall cell lung cancer treated with a mean dose of 20 Gy with a range of 15–25 Gy in a single fraction, pain improved in 89% of patients and the local control rate was 100% [40]. In addition with a followup of 12 months, no acute or chronic radiation toxicities were noted despite treatment being given to a mean volume of 25.7 cc (range: 0.2–264 cc). In the spine radiosurgery series with the longest median followup of 37 months, Gerszten et al. demonstrated similar efficacy of this treatment for renal cell carcinoma metastasis in 48 patients with little observed toxicity [41]. Again, a mean dose of 20 Gy in a single fraction (range: 17.5–25) was used to treat relatively large volumes (mean: 61.9 cc, range: 5.5–203 cc). Finally, in the largest published spine radiosurgical series to date consisting of 393 patients with a range of histologies, Gerszten and colleagues found, with a median followup of 21 months, that they achieved 88% tumor control and excellent palliation of pain with a mean dose again of 20 Gy (range: 12.5–25 Gy) [44]. Based on these series, spinal radiosurgery appears to be feasible, safe, and effective for the treatment of spinal metastatic disease of various histologies. 

In another series, Yamada and colleagues reported the Memorial Sloan-Kettering experience for spinal radiosurgery [45]. Here, 93 patients were treated to a median dose of 24 Gy (range: 18–24 Gy) with the spinal cord constrained to maximal point dose of 14 Gy. With a median followup of 15 months, the actuarial 1-year local control rate was 90% and, despite the relatively high single fraction dose of radiation, no myelopathy or other late toxicities were seen. Because a range of doses was used in this cohort, the impact of radiation dose on tumor control could be evaluated. This analysis revealed a dose-response relationship with higher doses being a statistically significant predictor of local control. 

In a phase I/II trial conducted at the MD Anderson Cancer Center by Chang et al., 63 patients underwent a hypofractionated course of spinal radiosurgery to a median tumor volume of 37.4 cc (range: 1.6–358 cc) [47]. Treatment given with a fractionation schedule of 6 Gy delivered in 5 fractions to half of the patients in the series that was later modified to 9 Gy delivered in 3 fractions to further reduce treatment time. With a median followup of 21 months, the one-year actuarial progression-free rate was 84%. The pattern of failure tended to be marginal being either in the bone adjacent to the site of previous treatment or in the epidural space adjacent to the spinal cord. No grade 3/4 neurologic toxicity was reported. Based on the pattern of failure in the posterior elements, the authors recommended inclusion of the pedicles and the posterior elements of the vertebrae in the target volume due to the possibility of direct extension to these structures. 

Ryu and colleagues at Henry Ford Hospital published a series consisting of 177 patients treated with single fraction radiosurgery with doses ranging from 8–18 Gy [32]. With a relatively short median followup of 6.7 months, they demonstrated that a dose to 10% of the spinal cord of 9.8 Gy was well tolerated with respect to acute toxicity. Of note, in the subgroup of eighty-six patients that survived more than 1 year, one case of spinal cord injury at 13 months after radiosurgery was seen. One conclusion of this series was that the tolerance of the spinal cord is at least 10 Gy to 10% of the cord volume as defined as 6 mm above and below the target lesion. While efficacy outcomes was not reported on the above study, this group published a followup paper showed excellent pain palliation with 41 of 49 patients who had significant pain prior to the procedure subsequently reporting on reduction in discomfort [48]. 

Nelson et al. described their clinical experience at Duke University for the treatment of spinal and paraspinal tumors in 32 patients with 33 spinal lesions [49]. In this series, the safety and efficacy of spinal radiosurgery was again demonstrated with a median followup of 7 months. Among the treated patients, 94% had improved pain control with 40% describing complete resolution of their pain. Moreover, no radiation-induced toxicity was observed. Interestingly, the authors used BED as calculated using the linear-quadratic model with a spinal alpha/beta ratio of 3 to define strict spinal cord limits in patients that had prior RT. Additionally, they utilized a model involving time-discounted BED recovery of the spinal cord based on prior published data [33]. Specifically, a dose recovery of 25%, 33%, and 50% at 6 months, 1 year, and 2 years, respectively, was accounted for in previously irradiated patients. While the authors conclude that spinal radiosurgery appears effective and safe when performed as prescribed, they caution that the time-discounted BED model of recovery will require further validation. 

Gibbs and colleagues at Stanford University reported on their series of 74 patients with 102 spinal metastasis treated with SBRS. Like the multiple other series reviewed here, they found that a high percentage of their patients (84%) had symptom improvement with an acceptable rate of toxicity [50]. A more recent report of the Stanford experience by Choi et al. focused on the safety and efficacy of spinal radiosurgery after previous irradiation [51]. Their series included 41 previously irradiated patients with recurrent metastatic spine disease. SBRS was delivered to the spine at a median marginal dose of 20 Gy in 2 fractions (range: 1–5 fractions). With a median followup of 7 months, the actuarial local control rate at 6 months and 1 year was 87% and 73%, respectively. Time to retreatment of less than or equal to 12 months was a significant predictors of local failure. While overall, the radiosurgery appeared to be well tolerated, one patient with metastatic breast cancer did develop a grade-4 neurotoxicity. At 81 months prior to retreatment, this patient had received a fractionated course of radiation (39.6 Gy in 1.8 Gy fractions) from T4 to L1 for spine disease resulting in a cord dose of 40 Gy. SBRS consisted of 20 Gy in 2 fractions to a 10.3 cc volume for a T5 recurrence with a maximum cord dose of 19.25 Gy. After experiencing LE weakness, paresthesias, and urinary retention 6 months after SBRS, the patient was diagnosed with a spinal cord injury, initiated on aggressive management without success, and ultimately became wheelchair dependent. Here, the authors also applied a time-discounted BED method, again extrapolating from Ang data similar to that used by Nelson et al. for choosing cord tolerances [33, 49, 51]. Similar to the other reports, they conclude that SBRS can be safely and effectively delivered for the treatment of spinal metastasis in previously irradiated regions. 

Degen et al. at Georgetown University published a series on 51 patients with 72 lesions that focused on pain control and quality of life assessments [52]. The visual analogue scale (VAS) and the 12-item Short Form Health Survey (SF-12) prior to and after treatment were used to assess these factors. In this cohort, the average VAS score decreased significantly from 51.5 to 21.3 at 4 weeks to 17.5 at 1 year indicating a very good initial reduction in pain that remained durable. Also, average SF-12 scores did not vary in either the physical or mental well-being domains over time, indicating quality of life maintenance after treatment. Gagnon et al. reported results of the followup study of similar design where this cohort was expanded to 200 patients and confirmed earlier results with respect to control of pain and maintenance of quality of life [53]. Overall, these studies were able to more objectively quantify the improvement in pain provided by radiosurgery and contribute to the growing body of evidence regarding the durability of response. 

Sahgal et al. reported on the results of spinal radiosurgery in 39 consecutive patients (with 60 tumors) at UCSF [54]. The median followup of patients in this study was 8.5 months. The median total dose prescribed was 24 Gy given in 3 fractions. Overall, the 1-year and 2-year progression-free probability was 85% and 69%, respectively. Of note, the great majority of failures had tumors that were less than or equal to 1 mm from the thecal sac. Finally, of the tumors followed for longer than 6 months (39 of 60), no radiation-induced neurotoxicity was noted. This study gives further support for the safety and efficacy of spinal radiosurgery. 

Pooling of the case series presented in this review results in a total of 1388 patients with 1775 lesions who underwent spinal radiosurgery. The combined result of these treatments is summarized on Table 2. The weighted (based on number of patients in each series) mean value of the median followup times for patients on all the series was slightly more than 15 months. In the series where pain relieve was examined, 79% of patients (*n* = 902) experienced some reduction in discomfort associated with their spinal lesions. The weighted overall local control rate, defined as lack of progression of the gross disease on surveillance imaging, was 90%. These results were obtained with an extremely low crude incidence of myelopathy of less than 0.5%. In summary, pooling the results of these case series further illustrates that spinal radiosurgery is a safe and effective treatment modality when performed as outlined by the various cited authors.

## 6. Clinical Recommendations

Numerous published series have now reported the results collectively on significantly more than 1000 patients treated with radiosurgery for spine metastatic disease thus establishing this treatment modality as a safe and effective therapy. However, certain standards need to be established to assure that treatment results are in line with what has been reported to date. We recommend that the procedure be a collaborative effort between the spine surgeon (neurosurgery or orthopedics) and the radiation oncologist with strong medical physics support. Since this procedure has many intricacies including issues with patient immobilization, treatment planning, accurate positioning, and so forth, it should be performed only at institutions that have made the commitment to establish a program that will see and treat a reasonable number of patients (e.g., >25 patients/year) in order to maintain proficiency with this procedure. Adequate quality assurance specific for the radiosurgery system used needs to be performed by the physics staff on a regular basis to assure that the equipment is performing according to specification. 

Patients need to be carefully selected, and informed consent obtained regarding the risks, benefits, and alternatives to spinal radiosurgery. Conventionally fractionated RT should be presented to the patient as a viable alternative to radiosurgical treatment. The site of treatment should be limited, and we recommend that disease involvement be at two or less contiguous vertebra(e). Based on the literature, a dose of 15–20 Gy delivered in a single fraction should be safe and effective. The spinal cord should be constrained so that no more than 10% of the cord, defined to include the target level and 6 mm above and below this region, receives 10 Gy. This dose constraint should be achievable in the great majority of cases unless there is epidural disease that is <3 mm from the edge of the spinal cord. In such cases, a hypofractionated approach utilizing between 2–4 treatment fractions to deliver 18–24 Gy may still be possible depending on spinal cord dosimetric considerations. Patients with frank cord compression, spinal instability secondary to compression fracture, or bony retropulsion causing neurologic symptoms should be considered for surgery, if possible.

## 7. Summary

The role of radiation therapy for the treatment of spinal tumors, whether metastatic or primary, is well established. While conventional radiation therapy delivered without the use of high-precision localization techniques has been used for decades [6, 7, 55, 56], over the past fifteen years, new radiotherapy technologies now enable the delivery of high doses of focal radiation therapy with steep dose fall-off and millimeter accuracy in sites other than the brain. The safety and efficacy of these new technologies for use in spinal tumors have been increasingly demonstrated. The concept behind spinal radiosurgery is extrapolated from the long-standing experience of radiosurgery in the brain as a treatment modality [57–60]. Given the majority of tumors in this review series were metastatic in nature; spinal radiosurgery should be considered as an emerging essential part of the treatment armamentarium for spinal metastatic disease. 

As illustrated in this review, numerous recently published series have shown that spinal radiosurgery can be given with high probability of tumor control and symptom relieve with a correspondingly low incidence of long-term toxicities. This treatment relies on high precision and highly conformal radiation doses delivered in 1–5 fractions, often very close to the spinal cord. It is important to note that each of the reviewed series is at a larger academic center that can perform this type of procedure at high volume and with adequate quality assurance. Application of these techniques at smaller radiotherapy centers where procedure volume will be lower and less physics/technical support is available should be approached with caution. 

Several questions remain about the application of the spinal radiosurgery procedure. These include the precise definition of the dose tolerance of the spinal cord at radiosurgical doses, the influence of fraction number when giving high-dose, multifraction treatments, the most effective dose schedule to use with respect to symptom reduction and tumor control, and how spinal radiosurgery compares with more conventional radiation therapy treatments for safety and efficacy. Based on the case series presented in this review, a dose of nearly 21 Gy delivered in an average of 1.6 fractions can be safely delivered with rates of myelopathy of less than 0.5% and results in excellent rates of tumor control and pain relief. Overall, the future of spinal radiosurgery continues to evolve. With an increasing number of new heavy particle accelerators, proton-based spinal radiosurgery may be increasingly considered. Clearly, proton-based therapy for spinal tumors will have several dosimetric advantages when compared to traditional photon-based techniques [61]. These dosimetric advantages could potentially result in even lower toxicity and risk associated with the spinal radiosurgery procedure. However, there is currently a lack of high-quality evidence to support or refute the clinical applicability of the dosimetric advantages that protons may provide. Finally, as stated above, spinal radiosurgery has not yet been compared to more conventional radiation therapy techniques in the prospective setting. This question is now the subject of the current cooperative group trial (RTOG 0631) examining 8 Gy in a single fraction to a wider field compared with 16 Gy in a single fraction to a more limited radiosurgical volume for spinal metastatic disease. Results of this trail are awaited with anticipation.

## 8. Conclusion

Stereotactic body radiosurgery for spinal tumors is increasingly assuming a larger role in the treatment of metastatic spinal lesions. It has been shown in numerous prospective cohort series and retrospective case series that spinal radiosurgery is both safe and effective. In addition, the minimally invasive nature of spinal radiosurgery and its ability to be performed on an outpatient basis lends itself extremely well to the patient population in which it is most frequently used. Because patients with metastatic disease to the spine often have large burdens of systemic disease and poor performance status, radiosurgery provides them with an attractive option to relieve their suffering quickly with very low risk.

## Figures and Tables

**Table 1:** Spine radiosurgical series through December 2010.

Authors and year	Institution	No. of pts/ tx sites	Prior RT	Treatment system	Dose/fraction No./coverage	Spinal cord dose limits	Histology	Median F/U (in months)	Local control rate (percent)	Pain improved (percent)	Rate of myelopathy (percent)
Amdur et al., 2009 [39]	U of Florida	21/25	12/21	In house (Elekta Synergy-S)	15 Gy/1/100% to 95% of PTV	12 Gy to < 0 .1 cc, 5 Gy to < 0.5 cc if prior RT	Many	8	24/25 (96%)	6/14 (43%)	0/21 (0 %)
Gerszten et al., 2006 [40]	U of Pittsburgh	77/87	70/77	CyberKnife	20 Gy (mean)/1/80% IDL	9 Gy max (mean), range: 4–12 Gy	Lung	16	87/87 (100%)	65/73 (89%)	0/77 (0%)
Gerszten et al., 2005 [41]	U of Pittsburgh	48/60	42/48	CyberKnife	20 Gy (mean)/1/80% IDL	9.7 Gy max (mean), range: 2.4–14.0 Gy	Renal	37	54/60 (90%)	37/38 (97%)	0/48 (0%)
Gerszten et al., 2005 [42]	U of Pittsburgh	28/36	23/28	CyberKnife	21.7 Gy (mean)/1/80% IDL	>8 Gy to 0.3 cc (range: 0–0.7 cc)	Melanoma	13	26/28 (93%)	27/28 (96%)	0/28 (0%)
Gerszten et al., 2005 [43]	U of Pittsburgh	50/68	48/50	CyberKnife	19 Gy (mean)/1/80% IDL	13 Gy max dose	Breast	16	68/68 (100%)	55/57 (96%)	0/50 (0%)
Gerszten et al., 2007 [44]	U of Pittsburgh	393/500	344/500	CyberKnife	20 Gy (mean)/1/80% IDL	NR	>50% breast, lung, melanoma, renal	21	440/500 (88%)	290/336 (86%)	0/393 (0%)
Yamada et al., 2008 [45]	Memorial Sloan-Kettering	93/103	0/93	In house [46]	24 Gy (median)/1/92% IDL (average)	11.7 Gy max (median), range: 1.8–14 Gy	Many	15	90% (actuarial at 1 year)	NR	0/93 (0%)
Chang et al., 2007 [47]	MD Anderson	63/74	35/63	In house (Varian 21EX)	tx 1 : 6 Gy/5/80–90% of PTVtx 2 : 9 Gy/3/80–90% of PTV	10 Gy max in 5 fractions for tx 1 9 Gy max in 3 fractions for tx 2	Many (40% renal)	21	84% (actuarial at 1 year)	NR	0/63 (0%)
Ryu et al., 2007 [32]	Henry Ford Hospital	177/230	0/177	Brainlab Novalis system	8–18 Gy/1/90% IDL	9.2 Gy (mean) to <10% of cord volume	Many (25% breast)	6	NR	NR, 41/49 (84%) separate report [48]	1/177 (0.5%)
Nelson et al., 2009 [49]	Duke U	32/33	22/32	In house (Varian 21EX)	18 Gy (median)/3 (median)/	12 Gy to 1% of cord, BED <83 for retreatment	Many (31% renal)	7	29/33 (88%)	30/32 (94%)	0/33 (0%)
Gibbs et al., 2007 [50]	Stanford U	74/102	50/74	Cyberknife	16–25 Gy/1–5/77% IDL (mean), 98% of PTV	Max dose range 3–28 Gy in 1–5 fractions	>50% breast, lung, melanoma, renal	9	NR	52/62 (84%, includes other neuro sxs)	3/74 (4%)
Choi et al., 2010 [51]	Stanford U	42/51	42/42	Cyberknife	20 Gy (median)/2 (median)/77% IDL (median)	19.3 Gy max (median) in 1–5 fractions	>50% breast, lung	7	38/51 (75%)	15/23 (65%)	1/41
Degen et al., 2005 [52]	Georgetown U	51/72	38/72	Cyberknife	21 Gy (mean)/3.6 (mean)/71% IDL (mean)	11 Gy max (mean) to <1% of cord volume	Many (19% breast)	12	69/72 (96%)	37/38 (97.3%)	0/51 (0%)
Gagnon et al., 2009 [53]	Georgetown U	200/274	137/274	Cyberknife	21, 26.4 or 37.5 Gy/3, 3 or 5/75% IDL	NR	Many (18% breast)	12	NR	55/152 (36%) became pain-free	0/200 (0%)
Sahgal et al., 2009 [54]	UCSF	39/60	25/39	Cyberknife	24 Gy/3/67% or 60% IDL (no RT or prev. RT)	16.8 Gy or 12.8 Gy max (median) (no RT or prev RT)	Many	9	85% (actuarial at 1 year)	NR	0/39 (0%)

Abbreviations: pts, patients; tx, treatment; F/U, followup; RT, radiation therapy; Gy, Gray; cc, cubic centimeter; PTV, planning target volume; IDL, isodose line; NR, not reported; BED, biologically equivalent dose.

**Table 2:** Pooled results of spinal radiosurgery series.

Description	Values
Total patients	1388
Total lesions	1775
Patients with previous RT	888
Mean F/U time (months)	15
Pain improvement rate (*n* = 902)	79%
Local control rate (*n* = 1169)	90%
Myelopathy rate (*n* = 1388)	0.4%

Abbreviations: RT, radiation therapy; F/U, followup.
